# Comparative Transcriptomic and ceRNA Network Analyses of Non-Coding and Coding RNAs in Heads of *Apis mellifera* Workers from Queenright and Queenless Colonies

**DOI:** 10.3390/ijms27083426

**Published:** 2026-04-11

**Authors:** Yunchao Kan, Yanru Chu, Huixuan Shi, Zhaonan Zhang, Runqiang Liu, Zhongyin Zhang, Dandan Li, Huili Qiao

**Affiliations:** 1 School of Plant Protection and Environment, Henan Institute of Science and Technology, Xinxiang 453003, China; yckan@henu.edu.cn (Y.K.); liurunqiang1983@126.com (R.L.); zzy206@126.com (Z.Z.); 2 Henan Key Laboratory of Insect Biology, Henan International Joint Laboratory of Insect Biology, College of Life Science, Nanyang Normal University, Nanyang 473061, China; chuyr1124@163.com (Y.C.); shx112823@163.com (H.S.); lidannytc@126.com (D.L.); 3 College of Bee Science and Biomedicine, Fujian Agriculture and Forestry University, Fuzhou 350001, China; 000q825148@fafu.edu.cn

**Keywords:** honeybee, *Apis mellifera*, lncRNA, miRNA, ovary development

## Abstract

Emerging evidence indicates that non-coding RNAs (ncRNAs) play important regulatory roles in honeybee social behavior and development. However, the regulatory roles of ncRNAs in honeybees remain largely elusive. To systematically identify ncRNAs associated with queen-regulated ovary activation, we conducted whole-transcriptome sequencing on the heads of *Apis mellifera* workers from queenright and queenless colonies. Subsequent bioinformatics analyses were conducted to profile differentially expressed (DE) RNAs and construct potential regulatory networks. High-quality sequencing data provided a foundation for subsequent analyses. This transcriptome data yielded 3968 lncRNA transcripts, comprising 3146 known and 822 novel candidates, all of which exhibited typical structural features of lncRNAs. Comparative expression analyses revealed that 246 lncRNAs, 1439 mRNAs, and 10 miRNAs were differentially expressed. Comprehensive functional analyses indicated that the identified DElncRNAs potentially regulate sensory perception-related target mRNAs via cis-regulation, and coordinate metabolic and proteostatic reprogramming via trans-regulation to support the transition to reproductive activation in workers. Furthermore, a competing endogenous RNA network was constructed which integrated 74 DElncRNAs, 5 DEmiRNAs, and 36 DEmRNAs to predict their potential post-transcriptional interactions. Our findings highlight a comprehensive analysis of ncRNAs and mRNAs in worker heads, providing a foundation for functional validation of their roles in honeybee ovary development.

## 1. Introduction

As important pollinators of plants, honeybees directly influence global ecosystem biodiversity and the productivity of agricultural crops [1]. Beyond their vital role as pollinators, honeybees are distinguished by a sophisticated social organization. Key adaptive traits, including division of labor, hive defense, and advanced communication systems, are crucial for the survival and reproduction of the colony [2]. These processes are coordinated through an interplay among genetic factors, environmental cues, and chemical signals [3].

The collective behaviors of honeybees are controlled by different chemical signals called pheromones, which are secreted for communication among individuals within a hive. Pheromones, whether volatile or not, act as molecular messengers to control a variety of colony activities, such as recognizing the queen, maintaining worker discipline, organizing the food collection, and controlling reproduction [4]. Queen mandibular pheromone (QMP) is a mixture of fatty acid derivatives released from the queen, which suppresses worker ovarian development and promotes colony unity [5,6]. Alarm pheromone released by guard bees triggers collective defensive responses from the hive, whereas larval pheromone regulates nursing and foraging behavior of workers [7,8]. In *A. mellifera*, QMP is a blend of five compounds secreted from the queen’s mandibular glands. As a central regulator of social organization, QMP influences several key physiological and behavioral traits in honeybees—for example, suppressing ovarian maturation [6,9], eliciting retinue behavior [10], enhancing associative learning [11], facilitating nest-mate discrimination [12], and regulating the age at foraging onset [13]. In honey bee colonies, the absence of a queen triggers profound shifts in worker physiology, most notably the activation of ovarian development—a process that is tightly suppressed by queen pheromones under normal social conditions. Research has documented that, without queen-derived signals, a subset of workers initiates oogenesis, with some even laying unfertilized eggs [9]. This transition involves dynamic changes in gene expression, including upregulation of vitellogenin and other reproductive genes, alongside alterations in endocrine pathways such as juvenile hormone signaling [14,15]. However, previous research on ovarian activation has primarily focused on gene expression and regulation within the ovary, whereas the regulation of upstream regulatory factors in the head remains underexplored.

In recent years, non-coding RNAs (ncRNAs) have emerged as key regulators of gene expression and behavior in insects, acting through transcriptional, post-transcriptional, and epigenetic mechanisms to shape complex traits. Major types such as microRNAs (miRNAs) and long non-coding RNAs (lncRNAs) lack protein-coding potential, but exert extensive influence over cellular processes by modulating gene expression both transcriptionally and post-transcriptionally [16,17]. As post-transcriptional regulators, miRNAs recognize complementary sequences within mRNA 3′UTRs and silence target genes through endonucleolytic cleavage or translational inhibition, thereby modulating a wide range of biological processes [18]. The functions of miRNAs have been extensively investigated across many organisms. In honeybees, miRNAs have been found to regulate many biological processes, including development, behavioral plasticity, caste differentiation, olfactory learning, and memory [19,20,21,22]. Research has identified several honeybee miRNAs with distinct regulatory functions. For instance, miR-14, differentially expressed between queen and worker bees, likely regulates reproductive physiology, while miR-305 influences the division of labor [20,23,24]. Additionally, miR-2161 modulates larval survival and development via the juvenile hormone pathway; miR-282 and miR-278 are linked to the regulation of foraging behavior in *A. mellifera* [25,26]. However, our understanding of the specific role of miRNAs in mediating behavioral and physiological responses to the presence of the queen remains limited.

LncRNAs are broadly defined as transcripts exceeding 200 nt with little or no protein-coding capacity. They regulate gene expression across transcriptional, post-transcriptional, and epigenetic layers [27]. Although less studied than miRNAs in honeybees, recent transcriptomic analyses have identified thousands of lncRNAs in Apis species, many of which are differentially expressed during caste development or in response to environmental stimuli [28,29,30,31]. For instance, an lncRNA (LOC113219358) has been identified as a critical molecule in the immune network of *A. mellifera*, where it may regulate key immune responses via protein interactions [32]. In *Drosophila melanogaster*, lncRNAs are involved in the regulation of circadian rhythms and social behavior, suggesting that similar regulation mechanisms may also occur in bees [33,34,35]. A recent study demonstrated that expression of the lncRNA lncov1 correlates with reduced reproductive capacity in adult queens and workers, responding to key fertility-regulating cues [36].

While ncRNAs are known to function as important molecular regulators in various biological processes of honey bees, how queens modulate their expression in the worker head to govern ovary activation has received limited attention. RNA-sequencing is a powerful method to track global changes in transcriptomes, including both mRNAs and ncRNAs. Here, we performed whole-transcriptome RNA sequencing on heads of worker bees (*A. mellifera*) from queenright (QR) and queenless (QL) colonies. The identification of key ncRNAs and their target genes will help elucidate the molecular pathways underlying queen-regulated ovary activation. This study aimed to perform an integrated analysis to identify differentially expressed (DE) lncRNAs, mRNAs, and miRNAs in worker heads, and to predict potential regulators involved in ovary development in response to the queen. These results will enrich our understanding of ncRNA regulation in honey bee colonies and provide a foundation for future research on honey bee ovary activation and behavior regulation.

## 2. Results

### 2.1. Data Summary of the Whole-Transcriptome Sequencing

**Figure 1:**
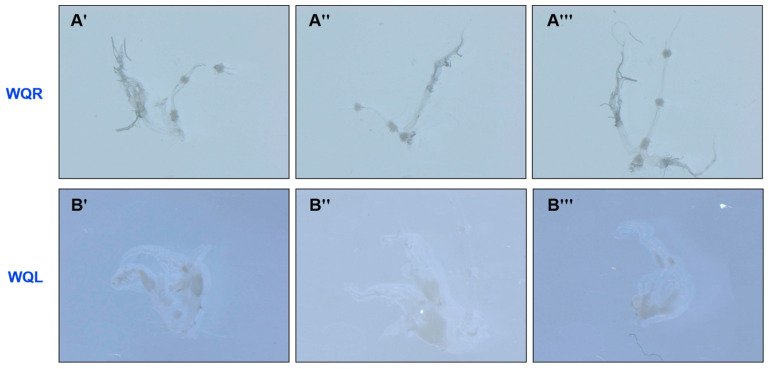
Representative ovary images of 14-day-old workers from queenright (QR) (**A′**–**A‴**) and queenless (QL) colonies (**B′**–**B‴**).

Ovary activation is regulated by the queen via queen pheromones [6]. To identify ncRNAs in worker heads that correlate with ovary activation, we first assessed the ovarian developmental status of 14-day-old workers from QR and QL colonies. Representative images of ovary phenotype are shown in Figure 1. The ovaries of workers from QL colonies were fully developed, whereas those from QR colonies remained undeveloped.

Based on these results and prior studies [37,38], six strand-specific cDNA libraries were constructed from the heads of 14-day-old queenright workers (WQR) and queenless workers (WQL). A total of 547 million raw reads (82.17 Gb) for lncRNAs and mRNAs were generated, and an average of 89 million clean reads (13.38 Gb) per sample were retained after quality control. All samples exhibited high sequencing quality, with Q30 scores exceeding 96.93%. The subsequent alignment of clean reads to the *A. mellifera* reference genome yielded mapping rates ranging from 80.02% to 95.26% across the six samples (Table 1). sRNA libraries were constructed from the same six samples and sequenced, yielding approximately 12 million raw reads (3.6 Gb) per sample. After quality filtering, an average of 11.9 million high-quality clean reads per sample was obtained, with Q30 scores exceeding 98.63%. Mapping efficiency ranged from 79.64% to 97.34%, confirming the high quality of the sRNA-seq dataset (Table 2).

### 2.2. Identification and Characteristics of Non-Coding and Coding RNAs

**Figure 2:**
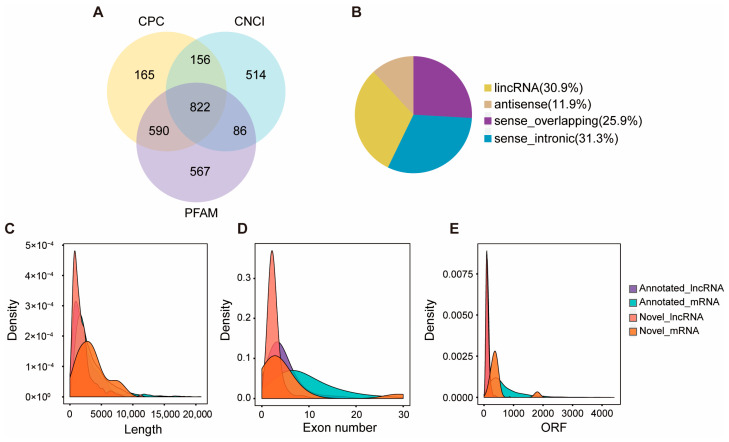
Identification and characteristic comparison of lncRNAs and mRNAs. (**A**) Identification of lncRNAs using CPC, CNCI, and PFAM. (**B**) Classification of the identified novel lncRNAs. (**C**) Distribution of transcript length density between lncRNAs and mRNAs. (**D**) Distribution of exon number density between lncRNAs and mRNAs. (**E**) Distribution of ORF length density between lncRNAs and mRNAs.

For lncRNAs, to distinguish protein-coding from non-coding transcripts, the coding potential of each novel lncRNA candidate was assessed using three tools: CPC, PFAM, and CNCI. Transcripts classified as non-coding by the three tools were retained. After stringent coding potential filtering, a final set of 3968 lncRNAs was obtained from the six samples, consisting of 3146 known lncRNAs and 822 novel lncRNAs (Figure 2A, Table S1). All novel lncRNAs were annotated and categorized into four distinct classes based on their genomic position relative to mRNAs: lincRNA (254, 30.9%), antisense (98, 11.9%), sense overlapping (213, 25.9%), and sense intronic (257, 31.3%) (Figure 2B). The structural features of lncRNAs and mRNAs were systematically compared based on transcript length, ORF length, and exon number. Compared to mRNAs, lncRNAs exhibited shorter average transcript length (2415 nt vs. 3708 nt) (Figure 2C, Table S1), fewer exons (4.09 vs. 10.3) (Figure 2D, Table S1), and shorter ORFs (126 nt vs. 814 nt) (Figure 2E, Table S1). The length of individual lncRNAs ranged from 111 to 23,523 nt.

For miRNAs, 176 known miRNAs were identified after data filtering and comparison with miRNA sequences in miRBase. The three samples of WQL yielded 155, 159, and 157 mature miRNAs, and the three samples of WQR yielded 156, 156, and 163 mature miRNAs. Additionally, 12 novel miRNAs were identified from the six samples using miREvo and mirdeep2 (Table 3).

### 2.3. Comparative Expression Profiling of Non-Coding and Coding RNAs

**Figure 3:**
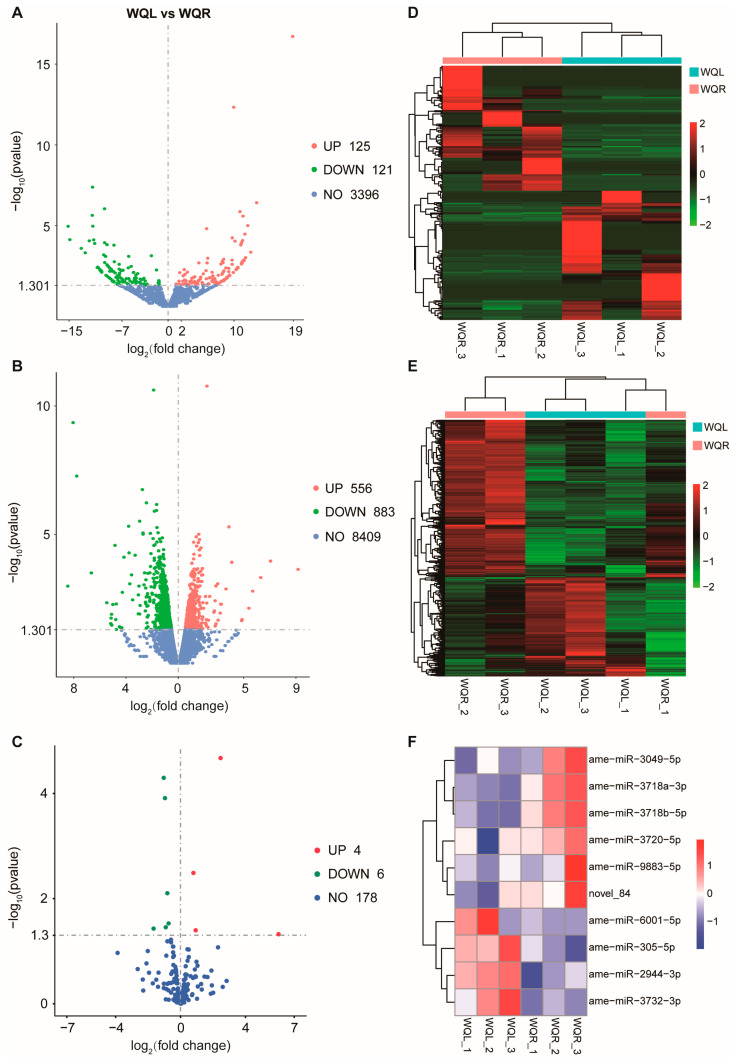
Volcano diagrams and Hierarchical cluster heatmap of DElncRNAs (**A**,**D**), DEmRNAs (**B**,**E**), and DEmiRNAs (**C**,**F**).

DElncRNAs, DEmRNAs, and DEmiRNAs were further identified between WQL and WQR groups, which yielded 246 DElncRNAs and 1439 DEmRNAs. Among these, the DElncRNAs comprised 125 up- and 121 down-regulated transcripts, while the DEmRNAs comprised 556 up- and 883 down-regulated transcripts (Figure 3A,B). Among the 188 miRNAs (176 known, 12 novel) identified in our study, we detected 10 DEmiRNAs, of which 4 miRNAs were up-regulated, and 6 miRNAs were down-regulated (Figure 3C). Hierarchical clustering heatmaps of differential transcripts are shown in Figure 3D–F.

### 2.4. Functional Analysis of DElncRNAs via Cis-Regulation

**Figure 4:**
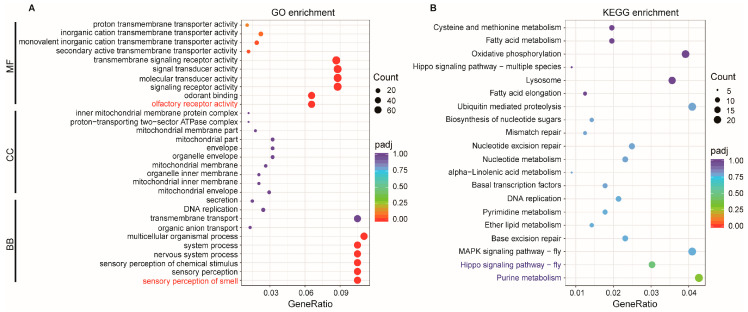
GO and KEGG enrichment analysis of cis-regulated target genes of DElncRNAs. (**A**) GO terms enriched among cis-regulated targets; (**B**) KEGG pathways associated with cis-regulated target genes. The most significantly enriched GO terms and KEGG pathways are highlighted in red and blue, respectively.

Accumulating studies have demonstrated that lncRNAs can influence the activity of nearby genes on the same chromosome, a mechanism defined as cis-regulation. Target genes regulated in this manner are referred to as cis-regulated target genes, also known as co-location genes. In this study, 3142 pairs of cis-regulated lncRNA-mRNA were predicted within a 100 Kb region, including 236 DElncRNAs and 2042 target genes. According to GO enrichment analysis, 60 genes were significantly overrepresented in six Biological Process (BP) terms, 121 genes in eight Molecular Function (MF) terms, and none in Cellular Component (CC) (Table S2). The top 10 GO terms in each ontology are presented in Figure 4A. The most significantly enriched terms were “sensory perception of smell” for Biological Process and “olfactory receptor activity” for Molecular Function, with no significant terms observed for Cellular Component. In parallel, KEGG enrichment analysis revealed that the predicted cis-target genes were significantly associated with “Purine metabolism” and “Hippo signaling pathway” (Figure 4B, Table S2).

### 2.5. Functional Analysis of DElncRNAs via Trans-Regulation

**Figure 5:**
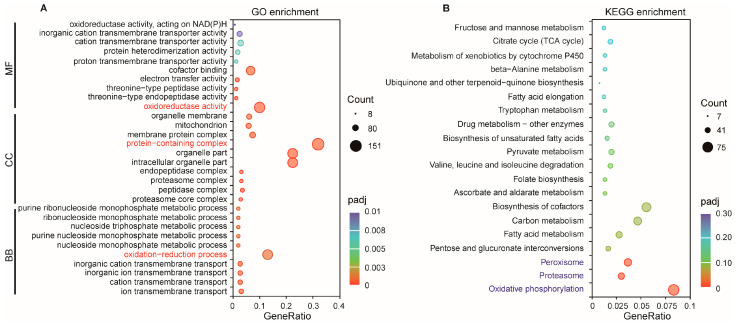
GO and KEGG enrichment analysis of trans-regulated target genes of DElncRNAs. (**A**) GO terms enriched among trans-regulated targets; (**B**) KEGG pathways associated with trans-regulated target genes. GO terms with the highest gene count and the most significantly enriched KEGG pathways are highlighted in red and blue, respectively.

LncRNAs can also directly regulate genes at long distances on the same or different chromosomes, which are known as trans-regulated target genes or co-expression genes. Here, 20,428 pairs of trans-regulated lncRNA-mRNA were predicted, comprising 204 DElncRNAs and 2696 target genes. GO annotation further revealed that 523 target genes were significantly enriched in 103 GO terms, comprising 53 Biological Process, 37 Cellular Component, and 13 Molecular Function terms (Table S3). The top 10 GO terms in each ontology are shown in Figure 5A. The GO terms with the highest number of enriched genes in the three ontologies were “oxidation-reduction process” for Biological Process, “protein-containing complex” for Cellular Component, and “oxidoreductase activity” for Molecular Function. KEGG pathway analysis showed that these trans-regulated targets participated in 117 kinds of metabolism or signal pathways, and the significantly enriched pathways were “Oxidative phosphorylation”, “Proteasome”, and “Peroxisome” (Figure 5B, Table S3).

### 2.6. Functional Analysis of DEmRNAs

**Figure 6:**
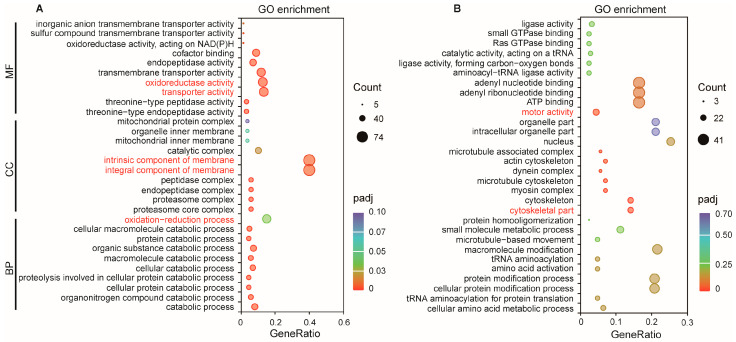
GO enrichment analysis of DEmRNAs. (**A**) Down-regulated mRNAs; (**B**) up-regulated mRNAs. GO terms with the highest gene count in (**A**) and the most significantly enriched GO terms in (**B**) are highlighted in red.

**Figure 7:**
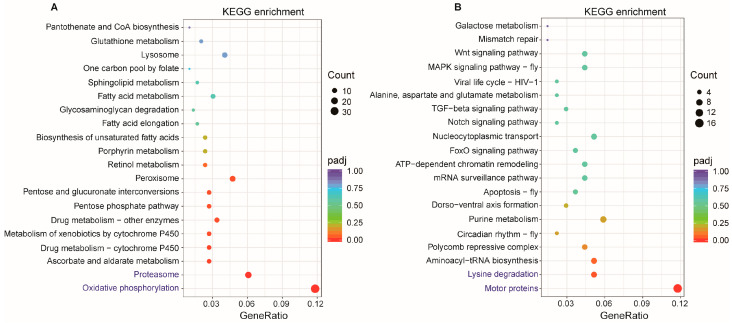
KEGG enrichment analysis of DEmRNAs. (**A**) down-regulated mRNAs; (**B**) up-regulated mRNAs. The most significantly enriched KEGG pathways are highlighted in Blue.

For DEmRNAs, 883 down-regulated mRNAs were enriched in 540 GO terms (Table S4). The top 10 GO terms in each ontology are shown in Figure 6A. A total of 12, 7, and 22 terms were significantly enriched in the categories of Biological Process, Cellular Component, and Molecular Function, respectively. The GO terms with high gene counts in the three ontologies were “oxidation-reduction process” for Biological Process, “component of membrane” for Cellular Component, “transporter activity” and “oxidoreductase activity” for Molecular Function. A total of 556 up-regulated mRNAs were enriched in 403 GO terms (Table S4). The top 10 GO terms in each ontology are shown in Figure 6B. The most significant terms were “cytoskeletal part” in Cellular Component and “motor activity” in Molecular Function, while no significant terms were identified in Biological Process. KEGG pathway analysis showed that 107 pathways were enriched by down-regulated mRNAs, and 9 of them were significant. The most significantly enriched pathways were “Oxidative phosphorylation” and “Proteasome”. While 70 pathways were enriched by up-regulated mRNAs, the most significantly enriched pathways were “motor proteins” and “lysine degradation” (Table S5). The top 20 pathways for both groups are shown in Figure 7A and Figure 7B, respectively.

### 2.7. CeRNA Networks of DElncRNAs, DEmRNAs, and DEmiRNAs

**Figure 8:**
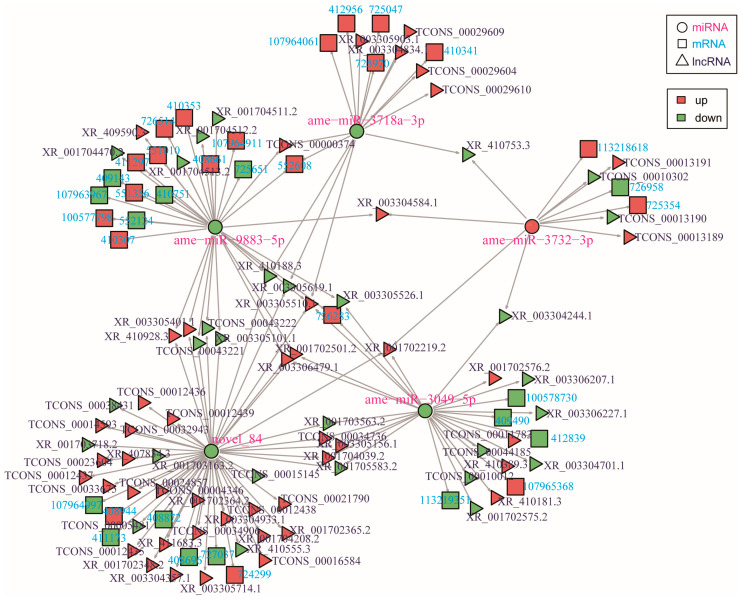
Regulatory network of DElncRNAs, DEmRNAs, and DEmiRNAs.

LncRNAs can competitively bind to miRNAs with their target mRNAs, inhibiting the repressive effect of miRNAs on these mRNAs and thus indirectly regulating gene expression. In this study, a total of 74 DElncRNAs were predicted to potentially target 5 DEmiRNAs, which were in turn predicted to bind to 36 DEmRNAs, suggesting a potential ceRNA network that warrants experimental validation. Among these, two lncRNAs (XR_001702501.2, XR_003306479.1) were predicted to target three miRNAs (ame-miR-3049-5p, ame-miR-9883-5p, novel_84). Additionally, 19 DElncRNAs (9 up-regulated, 10 down-regulated) were predicted to target two distinct miRNAs, whereas the remaining DElncRNAs each potentially targeted a single miRNA (Figure 8, Table S6).

### 2.8. Verification of DElncRNAs and DEmRNAs

**Figure 9:**
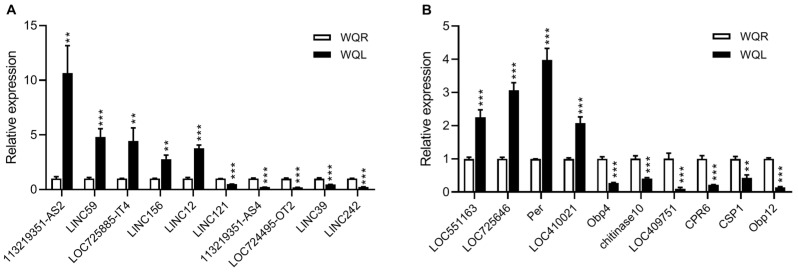
qPCR validation of selected DElncRNAs (**A**) and DEmRNAs (**B**). Data were presented as the mean ± standard deviation, and analyzed with two-tailed, unpaired *t*-test. ** *p* < 0.01, *** *p* < 0.001; significant differences are denoted above the bars.

To validate the RNA-seq results, 10 DElncRNAs (5 up-regulated and 5 down-regulated) and 10 DEmRNAs (4 up-regulated and 6 down-regulated) were randomly selected for qPCR detection. The up-regulated mRNAs included LMBR1 domain-containing protein 2 homolog (LOC551163), N-acetylneuraminate lyase (LOC725646), period circadian protein (Per), and protein skeletor isoforms B/C (LOC410021). The down-regulated mRNAs included odorant binding protein 4 (OBP4), chitinase10, multiple inositol polyphosphate phosphatase (LOC409751), cuticular protein 6 (CPR6), chemosensory protein 1 (CSP1), and odorant binding protein 12 (OBP12). qPCR validation confirmed that the expression patterns of these DElncRNAs and DEmRNAs were consistent with the RNA-seq data (Figure 9).

## 3. Discussion

To identify key molecules involved in queen-regulated ovary development, we constructed six strand-specific cDNA libraries and corresponding sRNA libraries from the heads of honeybee workers in queenright and queenless colonies for comprehensive transcriptomic analyses. The high Q30 scores and mapping rates for both lncRNA/mRNA and sRNA data indicate the high quality of the sequencing reads. Based on the transcriptome data, 3968 lncRNAs and 188 miRNAs were successfully identified. The 822 novel lncRNAs, together with 3146 known ones, greatly expand the existing lncRNA repertoire in honeybees. We categorized the novel lncRNAs into four types: lincRNAs (30.9%), antisense (11.9%), sense-overlapping (25.9%), and intronic-sense (31.3%), reflecting their distinct genomic origins and suggesting divergent functional roles [39]. These lncRNAs exhibited distinct structural features relative to mRNAs. LncRNAs had shorter mean lengths (2415 nt vs. 3708 nt for mRNA), fewer exons (4.09 vs. 10.3), and shorter ORFs (126 nt vs. 814 nt). This is consistent with the low protein-coding potential and reduced structural complexity characteristics of lncRNAs [40]. The identified lncRNAs follow common structural features but display considerable variation in their genomic loci.

It has been demonstrated that lncRNAs modulate the expression of target genes via cis- or trans-acting mechanisms. Here, we predicted 3142 cis-acting and 20,428 trans-acting lncRNA-mRNA regulatory pairs, highlighting the complexity of lncRNA-mediated regulatory networks. Cis-regulated genes were significantly enriched in GO terms related to olfaction. Worker bees rely on olfaction to detect queen pheromones, which are crucial for maintaining colony cohesion and reproductive division of labor. Pheromone detection occurs through specialized olfactory receptors in the antennae, with signal processing occurring in the brain [41]. The enrichment of “sensory perception of smell” and “olfactory receptor activity” among predicted lncRNA targets suggests that these lncRNAs may modulate worker sensitivity to queen pheromones. Previous study has shown that differentially expressed lncRNAs and mRNAs in the heads of nurse and forager bees are involved in sensory perception, including olfactory receptor activity and odorant binding [42]. This indicates that lncRNAs play a crucial role in regulating behavioral transition in honeybees. In our study, queen absence triggered a shift from a sterile to ovary activation in workers. LncRNAs may orchestrate this shift by regulating the expression of olfactory genes, thereby fine-tuning the worker’s response to the queen’s suppressive signals. KEGG analysis revealed that cis-regulated targets were significantly associated with “Purine metabolism” and “Hippo signaling pathway”. These pathways are critical for maintaining cellular energy balance and mounting stress responses, which are physiological capacities fundamental to worker task performance. Activating ovaries and preparing to lay eggs is an energetically expensive process [43]. A recent study on *Apis cerana* queens identified purine metabolism as the dominant ovarian pathway during activation, with gut microbiota potentially modulating ovarian morphology through purine signaling [44]. Thus, the enrichment of purine metabolism among the cis-target genes suggests that the foundational metabolic pathways required for ovary activation are not only activated in the ovaries themselves but are also transcriptionally primed or regulated in the heads of workers. The hippo signaling pathway is a highly conserved regulator of organ size, cell proliferation, and apoptosis [45]. In honey bees, a transcriptome-wide analysis of queen ovaries revealed that differentially expressed RNAs are related to Hippo, MAPK, Notch, and Wnt pathways during different phases of oviposition [46]. While these pathways are active in the ovary, it is intriguing that their upstream regulators (lncRNAs) are differentially expressed in the heads of workers from queenright and queenless colonies. It suggests that brain perception of social status influences lncRNA expression, which targets genes in reproductive pathways. This provides a potential molecular link between the social environment (presence or absence of the queen) and the activation of a core genetic program (Hippo pathway) that controls ovarian function.

Meanwhile, trans-regulated genes of lncRNAs were primarily involved in “oxidation-reduction process”, “protein-containing complex” and “oxidoreductase activity”, and were significantly enriched in “Oxidative phosphorylation”, “Proteasome”, and “Peroxisome” pathways. These functions and pathways are related to energy metabolism and protein homeostasis, thereby supporting cellular energy balance and viability in worker bees [47]. Ovary activation requires substantial ATP production to fuel cell proliferation, oocyte maturation, and vitellogenin synthesis [48]. LncRNAs have been shown to regulate energy metabolism through trans-acting mechanisms. Silencing the lncRNA (LOC113219358) in honey bee brains significantly altered expression of genes involved in ATP synthesis and oxidative phosphorylation [32]. The proteasome is responsible for degrading damaged or misfolded proteins and for regulating the turnover of key signaling molecules. During oogenesis and ovary development, precise control of protein degradation is essential for cell cycle progression, differentiation, and the removal of regulatory proteins that inhibit reproductive maturation [46]. In honey bee queens, oxidative phosphorylation and proteasome pathways are dynamically regulated during ovary activation and oviposition [14]. LncRNAs that trans-regulate proteasome-related genes may influence the stability and availability of proteins critical for ovary activation. Together, while cis-regulated lncRNAs may modulate sensory perception of queen pheromones, trans-regulated lncRNAs likely coordinate the metabolic and proteostatic reprogramming that supports the transition to reproductive activation in workers.

In addition, lncRNAs can also serve as precursors for miRNAs [49]. Here, 176 known and 12 novel miRNAs were identified, but direct evidence of DElncRNAs as miRNA precursors was not explicitly analyzed. However, given that 12 novel miRNAs were detected, it is plausible that some novel lncRNAs may act as precursors, as observed in mammals, where lncRNAs contribute to miRNA biogenesis [50]. Sense-intronic lncRNAs are spatially associated with pre-miRNA regions, potentially serving as substrates for Dicer processing [51]. Novel lncRNAs, particularly sense intronic lncRNAs, may function as miRNA precursors, and their roles in miRNA biogenesis need to be further studied.

Functional analysis of DEmRNAs revealed that most of down-regulated mRNAs were primarily enriched in “Oxidative phosphorylation” and “Proteasome” pathways. Notably, “Oxidation-reduction process” and “Oxidoreductase activity” were identified as the common pathways enriched among the trans-regulated targets of lncRNAs and down-regulated mRNAs. Despite the high energetic demands of ovary activation, oxidative phosphorylation and proteasome-related genes were down-regulated in the heads of queenless workers. It is explained by tissue-specific metabolic reallocation. In insects, the head primarily functions as a sensory and regulatory center, integrating environmental and social signals to orchestrate systemic physiological changes, while the metabolic burden of reproduction falls mainly on the ovaries and fat body. Down-regulation of energy metabolism genes in the brain may represent an energy conservation strategy that redirects resources toward reproductive tissues. This phenomenon is similar to the metabolic trade-off observed in other insect. In bumble bee workers, high juvenile hormone (JH) titers down-regulated genes involved in protein biosynthesis and turnover in the brain, but up-regulated mitochondrial and metabolic pathway genes in the fat body [52]. This down-regulation is JH-dependent in bumble bees, although JH is not the primary gonadotropin in honeybees, queenless condition may activate alternative neuroendocrine pathways that similarly suppress brain metabolic genes while promoting ovarian development [53]. Moreover, when workers become reproductively active under queenless conditions, they adopt a nurse-like physiological state and do not engage in foraging [54]. Research has revealed that nurses and foragers differ in brain energy metabolism gene expression [55]. Specifically, genes involved in the digestion of sugars, proteins, and fatty acids are down-regulated in the heads of nurses compared to foragers [29]. Our qPCR validation confirmed that genes related to olfaction (OBP4, OBP12 and CSP1), and energy metabolism (inositol polyphosphate phosphatase) were significantly down-regulated in queenless workers. OBPs and CSPs are small soluble proteins that bind and transport hydrophobic odorants and pheromones across the sensillum lymph to olfactory receptors [56]. OBP12, which is highly expressed in *A. mellifera* foragers [57], was down-regulated in queenless worker heads. Thus, the head transcriptome reflects the transition from a sensory- to a reproduction-oriented regulatory state rather than the metabolic execution of reproduction itself. The whole worker head introduces tissue heterogeneity, which may dilute transcriptional signals related to neural or olfactory regulation due to contributions from non-neural tissues. Nevertheless, the transcriptomic profiles reported here represent integrated responses across multiple head tissues. Specifically, enrichment of DElncRNA target genes and differential expression of olfactory genes suggest that biologically relevant neural signals were captured despite this heterogeneity. However, the tissue-specific expression and functional characterization of the identified lncRNAs require further elucidation in future studies.

Meanwhile, up-regulated mRNAs were enriched in “motor proteins” and “lysine degradation” pathways. Motor proteins and cytoskeletal components are essential for intracellular transport, vesicle trafficking, and neuronal plasticity [58]. A recent study found that in time-trained foragers, motor protein domain genes are enriched in the small-type Kenyon cells of mushroom bodies during foraging anticipation [59]. Our qPCR validation showed that protein skeletor gene was up-regulated in queenless workers. Up-regulation of motor protein-related genes in the brain may represent a cellular mechanism enabling the brain to adapt to the absence of the queen and coordinate downstream reproductive or foraging events. Lysine degradation not only contributes metabolism through acetyl-CoA production but also participates in epigenetic regulation. Lysine-based histone modifications are active epigenetic regulatory mechanisms in insects that respond to physiological state changes [60]. In addition, period circadian protein (Per) is well-characterized in honey bees. It shows daily oscillation in the brain and is involved in locomotor activity rhythms, time-memory, and foraging schedule [61]. Ovary-activated workers have a physiological state opposite to that of foragers [54]. Therefore, the up-regulation of per in queenless workers is likely to suppress foraging-related circadian activity by altering clock gene expression patterns. LMBR1 domain-containing protein is involved in various biological processes, such as transporting cobalamin out of lysosomes, regulating insulin receptor internalization, and participating in cell migration [62]. The homolog in *Drosophila* functions as a positive modulator in ovarian germ-line stem cell self-renewal [63]. N-acetylneuraminate lyase is involved in sialic acid metabolism and recycling in mammals [64]. Although it is differentially expressed between hygienic and non-hygienic honey bee colonies, its function remains unclear [65]. Thus, the up-regulation of mRNAs in the heads of queenless workers suggests that ovarian activation is accompanied by coordinated changes in neural function.

Based on the competing endogenous RNA (ceRNA) hypothesis, lncRNAs may function as ceRNAs by competitively sequestering miRNAs, preventing them from binding and inhibiting translation of targeted mRNAs [66]. In this study, 74 DElncRNAs were predicted to interact with 5 DEmiRNAs to potentially regulate 36 DEmRNAs, forming a ceRNA network. The integrated regulatory network (lncRNA-miRNA-mRNA) showed interconnected modules in which the differentially expressed lncRNAs, miRNAs, and mRNAs may coordinately regulate cellular functions and biological processes. Similar mechanisms have also been described in *D. melanogaster*, where ceRNA circuits regulate immunity by toll signaling [67]. Notably, 9 up-regulated and 10 down-regulated lncRNAs were each predicted to interact with two distinct differentially expressed miRNAs, respectively, suggesting that these lncRNAs may act as hub regulators integrating multiple signaling pathways. This proposed ceRNA network provides a valuable resource for hypothesis generation and offers candidate targets for future functional investigations. Meanwhile, it is necessary to mention that the queenless state may introduce potential confounding factors beyond queen pheromones, such as colony stress, nutritional changes, or altered social dynamics. These factors can independently influence ovarian activation and gene expression profiles in worker bees [68]. To minimize the impact of nutritional variation, all colonies were maintained under identical feeding schedule, with free access to sugar water and pollen throughout the experiment. Additionally, we selected only 14-day-old workers with similar body weights and ovarian developmental status to reduce individual variability. However, we cannot completely rule out the possibility of stress-related physiological changes or behavioral alterations in queenless colonies. Despite these limitations, our study provides a valuable transcriptomic resource for understanding ovarian activation under natural queen loss conditions, and the identified ncRNAs and mRNAs represent promising candidates for further functional validation. Experimental perturbations are required to validate the predicted interactions and their biological relevance in the following studies.

## 4. Materials and Methods

### 4.1. Insect Rearing and Collection

Colonies of *A. mellifera* used in this experiment were maintained on the campus of Nanyang Normal University, China. To obtain known-age bees, sealed worker brood frames containing late-stage pupae were transferred to an incubator at 33 °C one day before adult emergence. Worker bees were collected 20 h after eclosion and individually labeled with different colors. Then, the labeled workers were placed into six bee colonies, three of which had a queen (queenright worker, WQR) and three without a queen (queenless worker, WQL). Ovaries of 14-day-old workers were dissected and examined for developmental status using optical microscope. Only worker bees with undeveloped ovaries from QR colonies and those with fully developed ovaries from QL colonies were used for sampling. A total of 100 heads of the 14-day-old labeled bees from one colony were dissected as one sample, and immediately frozen in liquid nitrogen until RNA extraction.

### 4.2. RNA Extraction, Library Construction, and Whole-Transcriptome Sequencing

Total RNA isolation of the WQL and WQR head samples collected from six different bee populations was performed using TRIzol (Invitrogen, Carlsbad, CA, USA). RNA integrity and purity were verified by measuring its concentration with a Nanodrop spectrophotometer (Thermo Fisher Scientific, Wilmington, DE, USA), an integrity check using agarose gel electrophoresis, and purity determination on an Agilent 5400 Bioanalyzer instrument (Agilent Technologies, Santa Clara, CA, USA). RNA-seq libraries were prepared according to the manufacturer’s instructions (Illumina, San Diego, CA, USA) and sequenced on a NovaSeq platform at Novogene Bioinformatics Institute (Beijing, China).

For lncRNA library construction, rRNAs were depleted from the whole RNA samples by using specific probes, and the enriched mRNAs and ncRNAs were fragmented by heating in the presence of a divalent ion for fragmentation. After reverse transcription, the synthesized cDNA was subjected to end repair, A-tailing, and adapter ligation. PCR enrichment was performed after fragment selection. PCR products were purified to obtain a strand-specific library.

For sRNA library construction, small RNAs were ligated with specific adaptors at their 3′-end (3′-adaptor) and 5′-end (5′-adaptor). After ligation, cDNA was amplified using an RT primer. Then, the obtained cDNA was further amplified by PCR to generate a double-stranded DNA library. The libraries were purified and size-selected, retaining only those with insert length ranging from 18 to 40 bp before sequencing.

After library construction, the insert fragment size of each library was determined using an Agilent 2100 Bioanalyzer (Agilent Technologies, Santa Clara, CA, USA), and the effective concentration was quantified by real-time PCR. Libraries that met the quality criteria were normalized, pooled in equal molar amounts, and sequenced on an Illumina HiSeq platform according to the required effective concentration and data output.

### 4.3. Raw Data Analysis

Raw FASTQ files were first processed using fastp (v0.23.1) to trim adapter sequences, remove low-quality bases, and filter out reads containing ambiguous nucleotides (N). Following preprocessing, high-quality sequence data were obtained. The quality of each clean dataset was assessed by calculating standard metrics (Q20, Q30, and GC content). These quality-verified datasets served as the primary input for all subsequent bioinformatics analyses. For transcriptome alignment, clean reads from the lncRNA dataset were aligned with the *A. mellifera* genome using Hisat2 (v2.0.5) to obtain the location information of reads on the genome [69]. Bowtie (v1.0.1) is a short sequence comparison software to locate sRNA on the reference genome of the honeybee, allowing for the genome-wide distribution of sRNA to be mapped and visualized [70].

### 4.4. Identification and Characterization of lncRNAs

Sequencing reads from the pooled samples were assembled using Stringtie (v1.3.3), and the transcripts whose chain direction was uncertain and length less than 200 nt were removed [71]. The filtered transcripts were compared to existing references using Gffcompare (v0.10.6), which allows for the identification and removal of previously annotated transcripts. The other new transcripts are predicted for coding potential using the CPC2 (v3.2.0) [72], PFAM (v1.6) [73], and CNCI (v2.0) [74]. The predicted novel lncRNAs were classified based on their genomic locations relative to known protein-coding genes. In addition, a comprehensive structure comparison of lncRNA with mRNA was performed based on the transcript sizes, exon and intron numbers, as well as exon and intron lengths.

### 4.5. Identification of miRNAs

The sequenced reads that matched the reference genome were mapped onto a selected miRBase sequence database using mirdeep2 (v2.0.1.3) software. All available information about the known miRNAs, including structure, sequence, size, and abundances was obtained. Putative novel miRNAs were predicted using the characteristic stem-loop structure of miRNA hairpin precursors. After removing rRNA, tRNA, snRNA, snoRNA, or any other type of ncRNA as well as duplicate reads, novel miRNA candidates were predicted using miREvo (v1.1) [75] and mirdeep2 (v2.0.1.3) [76].

### 4.6. Differential Expression Analysis of Coding and Non-Coding RNAs

Mapped, spliced, filtered transcripts and computationally predicted transcripts were quantified using Stringtie (v1.3.3). The expression level of each transcript was quantified as Fragments Per Kilobase per Million mapped reads (FPKM) for each lncRNA or mRNA. Subsequently, the edgeR package (v3.22.5) was used to perform differential expression analysis between sample groups [77]. We used Transcripts Per Million (TPM) to quantify miRNAs and calculated differential miRNAs using DESeq2 (v1.42.0) [78]. To obtain statistically significant enrichment analysis results, we adopted a relaxed threshold of *p* < 0.05 (uncorrected) for initial screening, while an absolute fold change greater than 2 (|log2(FC)| > 1) was used as an additional filter.

### 4.7. Target Gene Prediction and Functional Enrichment

LncRNAs regulate target gene expression at both transcriptional and post-transcriptional levels. To identify the differential functional pathways generated by different treatments, the co-location (cis) and co-expression (trans) target genes of differentially expressed lncRNAs were predicted following the previous method [79,80]. The cis-targeted genes were searched within 100 kb upstream or downstream of all the identified lncRNAs, and their function was analyzed by functional enrichment. The trans-targeted genes were analyzed according to the co-expression relationships of lncRNAs and mRNAs. Pearson’s correlation coefficients between the expression levels of lncRNAs and mRNAs were calculated with custom scripts (r > 0.95 or r < −0.95), and the candidate trans-target genes were used for functional enrichment analysis. Target prediction of miRNAs was performed with miRanda (v3.3a) with strict seed region complementarity and a default energy threshold of −10 kcal/mol [81]. Enrichment of GO terms and KEGG pathways among DEmRNAs, DElncRNA target genes, and DEmiRNA target genes was assessed using clusterProfiler (v4.8.1) [82]. The significance threshold for enrichment was set at adjusted *p* < 0.05.

### 4.8. Construction of lncRNA-miRNA-mRNA Network

DEmiRNA-targeted DElncRNAs and DEmRNAs were predicted using miRanda (v3.3a) with strict seed region complementarity and a default energy threshold of −10 kcal/mol [81]. To elucidate the underlying ceRNA regulatory relationships, we constructed an integrated interaction network among the identified DElncRNAs, DEmiRNAs, and DEmRNAs. A ceRNA pair was defined as a DElncRNA and a DEmRNA that shared at least one DEmiRNA with which both exhibited a negative expression correlation. In this model, lncRNAs act as miRNA sponges, sequestering DEmiRNAs and thereby leading to the derepression of their target DEmRNAs. The network was built based on computationally predicted RNA–RNA interactions and visualized using Cytoscape (v3.10.1) [83].

### 4.9. Quantitative Real-Time PCR Validation

To verify the reliability of the transcriptome data used in this study, 10 DElncRNAs and 10 DEmRNAs were randomly selected for Quantitative real-time PCR (qPCR) validation. Total RNA from six honeybee heads samples were used for reverse transcription to synthesize the first strand cDNAs, which were then used for qPCR detection. The 10 μL reaction system was prepared using FastStart Universal SYBR Green Master (Rox) (Roche Diagnostics GmbH, Mannheim, Germany) following to the manufacturer’s instruction, and conducted on a Bio-RAD Real-time PCR instrument (Bio-Rad Laboratories, Hercules, CA, USA). The relative expression level of DElncRNAs and DEmRNAs were calculated using honeybee *Rpl32* gene (GeneBank ID: NM_001011587.1) as the internal reference with 2^−∆∆Ct^ method [84]. Specific primers for the selected DElncRNAs and DEmRNAs were designed with NCBI primer designing tool and synthesized by Sangon Biotech Co., Ltd. (Shanghai, China) (Table S7). All the data were plotted using GraphPad Prism 5.0, and the statistical analysis was conducted using two-tailed, unpaired *t*-tests to assess the significant differences. The data were shown as the mean ± standard deviation (SD) of three independent experiments.

## 5. Conclusions

This study provides a comprehensive transcriptomic landscape of lncRNAs, mRNAs, and miRNA in worker heads of *A. mellifera* associated with queen-regulated ovary activation. A total of 3968 lncRNAs and 188 miRNAs were identified, with 246 DElncRNAs, 1439 DEmRNAs, and 10 DEmiRNAs detected between WQL and WQR groups. Integrated analysis revealed that cis-regulated lncRNA targets were enriched in olfactory and Hippo signaling pathways, indicating potential functions in queen pheromone perception and reproductive control. In contrast, trans-regulated targets are enriched in energy metabolism and proteostasis, suggesting that lncRNAs coordinate metabolic reprogramming to support ovarian activation. A ceRNA network involving 74 lncRNAs, 5 miRNAs, and 36 mRNAs was also constructed, providing candidate interactions for future validation. Together, these findings reveal that lncRNAs act as critical molecular links between social environment and reproductive physiology in worker bees, offering a foundation for functional studies on the regulatory mechanisms underlying ovary activation.

## Figures and Tables

**Table 1:** Summary of the strand-specific cDNA library sequencing in the whole-transcriptome.

Samples	Raw_Reads	Total Reads	Mapped Reads	Q30 (%)
WQL_1	93,163,850	90,929,070	83,099,729 (91.39%)	97.28
WQL_2	85,496,558	83,846,738	67,093,838 (80.02%)	96.96
WQL_3	82,393,056	80,915,288	75,435,478 (93.23%)	96.93
WQR_1	93,145,662	90,970,748	86,659,407 (95.26%)	97.74
WQR_2	95,650,118	92,925,416	85,989,503 (92.54%)	96.96
WQR_3	97,979,758	95,508,356	88,028,256 (92.17%)	96.97

**Table 2:** Summary of the small RNA library sequencing in the whole-transcriptome.

Samples	Total Reads	Clean Reads	Mapped Reads	Q30 (%)
WQL_1	13,276,482	13,053,943	9,348,156 (96.60%)	98.77
WQL_2	13,773,061	13,666,357	10,342,671 (86.17%)	98.84
WQL_3	9,923,350	9,790,293	6,854,461 (79.64%)	97.89
WQR_1	11,382,423	11,217,681	8,184,710 (97.22%)	98.85
WQR_2	12,235,840	12,058,811	10,081,067 (97.31%)	98.84
WQR_3	11,651,499	11,442,013	9,866,839 (97.34%)	98.63

**Table 3:** Summary of miRNA identification.

Types	Total	WQL_1	WQL_2	WQL_3	WQR_1	WQR_2	WQR_3
mature miRNA	176	155	159	157	156	156	163
novel miRNA	12	7	10	9	8	7	9

## Data Availability

Sequence data that support the findings of this study have been deposited in the National Center for Biotechnology Information with the BioProject accession code PRJNA1423425.

## Supplementary Materials

The following supporting information can be downloaded at: https://www.mdpi.com/article/10.3390/ijms27083426/s1.

## Abbreviations

The following abbreviations are used in this manuscript:

LncRNA	Long non-coding RNA
miRNA	microRNA
QMP	Queen Mandibular Pheromone
WQR	Queenright Worker
WQL	Queenless Worker
GO	Gene Ontology
KEGG	Kyoto Encyclopedia of Genes and Genomes
ceRNA	Competing endogenous RNA
JH	Juvenile Hormone
OBP	Odorant Binding Protein
CSP	Chemosensory Protein
qPCR	Quantitative real-time PCR

## References

[1] Klein A.M., Vaissière B.E., Cane J.H., Steffan-Dewenter I., Cunningham S.A., Kremen C., Tscharntke T. (2007). Importance of pollinators in changing landscapes for world crops. Proc. Biol. Sci..

[2] Seeley T.D. (1995). The Wisdom Of the Hive: The Social Physiology of Honey Bee Colonies.

[3] Potts S.G., Biesmeijer J.C., Kremen C., Neumann P., Schweiger O., Kunin W.E. (2010). Global pollinator declines: Trends, impacts and drivers. Trends Ecol. Evol..

[4] Slessor K.N., Winston M.L., Le Conte Y. (2005). Pheromone communication in the honeybee (*Apis mellifera* L.). J. Chem. Ecol..

[5] Slessor K.N., Kaminski L.A., King G.G.S., Borden J.H., Winston M.L. (1988). Semiochemical basis of the retinue response to queen honey bees. Nature.

[6] Hoover S.E., Keeling C.I., Winston M.L., Slessor K.N. (2003). The effect of queen pheromones on worker honey bee ovary development. Naturwissenschaften.

[7] Sagili R.R., Pankiw T. (2009). Effects of Brood Pheromone Modulated Brood Rearing Behaviors on Honey Bee (*Apis mellifera* L.) Colony Growth. J. Insect Behav..

[8] Nouvian M., Reinhard J., Giurfa M. (2016). The defensive response of the honeybee *Apis mellifera*. J. Exp. Biol..

[9] Butler C.G., Fairey E.M. (1963). The Role of the Queen in Preventing Oogenesis in Worker Honeybees. J. Apicult. Res..

[10] Keeling C.I., Slessor K.N., Higo H.A., Winston M.L. (2003). New components of the honey bee (*Apis mellifera* L.) queen retinue pheromone. Proc. Natl. Acad. Sci. USA.

[11] Vergoz V., Schreurs H.A., Mercer A.R. (2007). Queen pheromone blocks aversive learning in young worker bees. Science.

[12] Karpe S.D., Dhingra S., Brockmann A., Sowdhamini R. (2017). Computational genome-wide survey of odorant receptors from two solitary bees *Dufourea novaeangliae* (Hymenoptera: Halictidae) and *Habropoda laboriosa* (Hymenoptera: Apidae). Sci. Rep..

[13] Robinson G.E., Winston M.L., Huang Z., Pankiw T. (1998). Queen mandibular gland pheromone influences worker honey bee (*Apis mellifera* L.) foraging ontogeny and juvenile hormone titers. J. Insect Physiol..

[14] Cardoen D., Wenseleers T., Ernst U.R., Danneels E.L., Laget D., DE Graaf D.C., Schoofs L., Verleyen P. (2011). Genome-wide analysis of alternative reproductive phenotypes in honeybee workers. Mol. Ecol..

[15] Robinson G.E., Strambi C., Strambi A., Huang Z.Y. (1992). Reproduction in worker honey bees is associated with low juvenile hormone titers and rates of biosynthesis. Gen. Comp. Endocrinol..

[16] Mercer T.R., Dinger M.E., Mattick J.S. (2009). Long non-coding RNAs: Insights into functions. Nat. Rev. Genet..

[17] Krol J., Loedige I., Filipowicz W. (2010). The widespread regulation of microRNA biogenesis, function and decay. Nat. Rev. Genet..

[18] Bartel D.P. (2009). MicroRNAs: Target recognition and regulatory functions. Cell.

[19] Chen X., Yu X., Cai Y., Zheng H., Yu D., Liu G., Zhou Q., Hu S., Hu F. (2010). Next-generation small RNA sequencing for microRNAs profiling in the honey bee *Apis mellifera*. Insect Mol. Biol..

[20] Ashby R., Forêt S., Searle I., Maleszka R. (2016). MicroRNAs in Honey Bee Caste Determination. Sci. Rep..

[21] Greenberg J.K., Xia J., Zhou X., Thatcher S.R., Gu X., Ament S.A., Newman T.C., Green P.J., Zhang W., Robinson G.E. (2012). Behavioral plasticity in honey bees is associated with differences in brain microRNA transcriptome. Genes. Brain Behav..

[22] Huang J., Wang T., Qiu Y., Hassanyar A.K., Zhang Z., Sun Q., Ni X., Yu K., Guo Y., Yang C. (2023). Differential Brain Expression Patterns of microRNAs Related to Olfactory Performance in Honey Bees (*Apis mellifera*). Genes.

[23] Chen X., Fu J. (2021). The microRNA miR-14 Regulates Egg-Laying by Targeting EcR in Honeybees (*Apis mellifera*). Insects.

[24] Stuart S.H., Ahmed A.C.C., Kilikevicius L., Robinson G.E. (2024). Effects of microRNA-305 knockdown on brain gene expression associated with division of labor in honey bee colonies (*Apis mellifera*). J. Exp. Biol..

[25] Song Y.X., Ren Y.P., Ran Y.Y., Fan N., Wu T., Zang H., Jiao M.X., Yan T.Z., Luo Q.M., Chen D.F. (2026). Ame-miR-2161 affects the survival and development of honeybee larvae through the juvenile hormone acid methyltransferase gene. Insect Mol. Biol..

[26] Li L., Liu F., Li W., Li Z., Pan J., Yan L., Zhang S., Huang Z.Y., Su S. (2012). Differences in microRNAs and their expressions between foraging and dancing honey bees, *Apis mellifera* L.. J. Insect Physiol..

[27] Rinn J.L., Chang H.Y. (2012). Genome regulation by long noncoding RNAs. Annu. Rev. Biochem..

[28] Humann F.C., Tiberio G.J., Hartfelder K. (2013). Sequence and expression characteristics of long noncoding RNAs in honey bee caste development--potential novel regulators for transgressive ovary size. PLoS ONE.

[29] Chen Y.J., Li Y.J., Wu S., Yang W.C., Miao J., Gu S.H., Li J.H., Miao X.Q., Li X. (2021). Transcriptional identification of differentially expressed genes associated with division of labor in *Apis cerana cerana*. Insect Sci..

[30] Wang Z., Wang S., Fan X., Zhang K., Zhang J., Zhao H., Gao X., Zhang Y., Guo S., Zhou D. (2023). Systematic Characterization and Regulatory Role of lncRNAs in Asian Honey Bees Responding to Microsporidian Infestation. Int. J. Mol. Sci..

[31] Zhang B., Zhang C., Zhang J., Lu S., Zhao H., Jiang Y., Ma W. (2024). Regulatory roles of long non-coding RNAs in short-term heat stress in adult worker bees. BMC Genom..

[32] Huang M., Tan X., Yang S., Zhou Z., Wang D., Dong J. (2025). Long Non-Coding RNA LOC113219358 Regulates Immune Responses in *Apis mellifera* Through Protein Interactions. Int. J. Mol. Sci..

[33] Soshnev A.A., Ishimoto H., McAllister B.F., Li X., Wehling M.D., Kitamoto T., Geyer P.K. (2011). A conserved long noncoding RNA affects sleep behavior in *Drosophila*. Genetics.

[34] Li M., Wen S., Guo X., Bai B., Gong Z., Liu X., Wang Y., Zhou Y., Chen X., Liu L. (2012). The novel long non-coding RNA CRG regulates *Drosophila* locomotor behavior. Nucleic Acids Res..

[35] Cui M.Y., Xu M.B., Wang Y.X., Bai B.Y., Chen R.S., Liu L., Li M.X. (2024). Long noncoding RNA LRG modulates *Drosophila* locomotion by sequestering Synaptotagmin 1 protein. Insect Sci..

[36] Cardoso-Júnior C.A.M., Tibério G.J., Peruzzolo M.C., Vieira L.C., Lago D.C., Paschoal A.R., Ronai I., Oldroyd B.P., Hartfelder K. (2026). A sterility-associated long noncoding RNA involved in honey bee caste determination and adult queen and worker fertility. Proc. Natl. Acad. Sci. USA.

[37] Villar G., Hefetz A., Grozinger C.M. (2019). Evaluating the Effect of Honey Bee (*Apis mellifera*) Queen Reproductive State on Pheromone-Mediated Interactions with Male Drone Bees. J. Chem. Ecol..

[38] Kuszewska K., Woloszczuk A., Woyciechowski M. (2024). Reproductive Cessation and Post-Reproductive Lifespan in Honeybee Workers. Biology.

[39] Derrien T., Johnson R., Bussotti G., Tanzer A., Djebali S., Tilgner H., Guernec G., Martin D., Merkel A., Knowles D.G. (2012). The GENCODE v7 catalog of human long noncoding RNAs: Analysis of their gene structure, evolution, and expression. Genome Res..

[40] Ponting C.P., Oliver P.L., Reik W. (2009). Evolution and functions of long noncoding RNAs. Cell.

[41] Paoli M., Galizia G.C. (2021). Olfactory coding in honeybees. Cell Tissue Res..

[42] Liu F., Shi T., Qi L., Su X., Wang D., Dong J., Huang Z.Y. (2019). lncRNA profile of *Apis mellifera* and its possible role in behavioural transition from nurses to foragers. BMC Genom..

[43] Harshman L.G., Zera A.J. (2007). The Cost of Reproduction: The Devil in the Details. Trends Ecol. Evol..

[44] Zhao C., Peng Y., Li W., Raza M.F., Wang W., Zhang Y., Chen Y., Guo J., Huang S., Han R. (2025). The role of gut microbiota-gonadal axis in ovary activation of Asian honey bee (*Apis cerana*) queens. npj Biofilms Microbiomes.

[45] Kim W., Jho E.H. (2018). The history and regulatory mechanism of the Hippo pathway. BMB Rep..

[46] Chen X., Ma C., Chen C., Lu Q., Shi W., Liu Z., Wang H., Guo H. (2017). Integration of lncRNA-miRNA-mRNA reveals novel insights into oviposition regulation in honey bees. PeerJ.

[47] Menail H.A., Cormier S.B., Léger A., Robichaud S., Hebert-Chatelain E., Lamarre S.G., Pichaud N. (2023). Age-related flexibility of energetic metabolism in the honey bee *Apis mellifera*. FASEB J..

[48] Han B., Wei Q., Amiri E., Hu H., Meng L., Strand M.K., Tarpy D.R., Xu S., Li J., Rueppell O. (2022). The molecular basis of socially induced egg-size plasticity in honey bees. eLife.

[49] Singh M., Priya K., Nagar A., Nalavade R. (2025). Emerging landscape of lncRNA-miRNA interactions as architects of gene expression patterns. Mol. Biol. Rep..

[50] Liz J., Portela A., Soler M., Gómez A., Ling H., Michlewski G., Calin G.A., Guil S., Esteller M. (2014). Regulation of pri-miRNA processing by a long noncoding RNA transcribed from an ultraconserved region. Mol. Cell.

[51] Wu Y., Cheng T., Liu C., Liu D., Zhang Q., Long R., Zhao P., Xia Q. (2016). Systematic Identification and Characterization of Long Non-Coding RNAs in the Silkworm, *Bombyx mori*. PLoS ONE.

[52] Shpigler H.Y., Herb B., Drnevich J., Band M., Robinson G.E., Bloch G. (2020). Juvenile hormone regulates brain-reproduction tradeoff in bumble bees but not in honey bees. Horm. Behav..

[53] Zhao M., Wu J., Kang W., Wei Q., Xu S., Guo H., Han B. (2026). Queen Loss Remodels Brain Dopamine and Hormonal Pathways During Worker Ovary Activation in *Apis mellifera*. Insects.

[54] Nakaoka T., Takeuchi H., Kubo T. (2008). Laying workers in queenless honeybee (*Apis mellifera* L.) colonies have physiological states similar to that of nurse bees but opposite that of foragers. J. Insect Physiol..

[55] Tan K., Wang Y., Dong S., Liu X., Zhuang D., Chen W., Oldroyd B.P. (2015). Associations between reproduction and work in workers of the Asian hive bee *Apis cerana*. J. Insect Physiol..

[56] Pelosi P., Iovinella I., Zhu J., Wang G., Dani F.R. (2018). Beyond chemoreception: Diverse tasks of soluble olfactory proteins in insects. Biol. Rev. Camb. Philos. Soc..

[57] Liu F., Li W., Li Z., Zhang S., Chen S., Su S. (2011). High-abundance mRNAs in *Apis mellifera*: Comparison between nurses and foragers. J. Insect Physiol..

[58] Nambiar A., Manjithaya R. (2024). Driving autophagy-the role of molecular motors. J. Cell Sci..

[59] Roy T., Jain R., Brockmann A. (2025). Transcriptional responses in feeder time-trained foragers suggest diverse interactions between the circadian clock and mushroom bodies in honey bees. Sci. Rep..

[60] Yu Z., Pei T., Wang H., Wang C., Liu J., Storey K.B. (2024). Lysine Methylation and Histone Modifications during Cold Stress of Insects: Freeze-Tolerant Eurosta solidaginis and Freeze-Avoiding Epiblema scudderiana. Insects.

[61] Abreu F.C.P., Freitas F.C.P., Simões Z.L.P. (2018). Circadian Clock Genes Are Differentially Modulated during the Daily Cycles and Chronological Age in the Social Honeybee (*Apis Mellifera*). Apidologie.

[62] Kelsey J.S., Fastman N.M., Blumberg D.D. (2012). Evidence of an evolutionarily conserved LMBR1 domain-containing protein that associates with endocytic cups and plays a role in cell migration in dictyostelium discoideum. Eukaryot. Cell..

[63] Dolezal D., Liu Z., Zhou Q., Pignoni F. (2015). Fly LMBR1/LIMR-type protein Lilipod promotes germ-line stem cell self-renewal by enhancing BMP signaling. Proc. Natl. Acad. Sci. USA.

[64] Sánchez-Carrón G., García-García M.I., López-Rodríguez A.B., Jiménez-García S., Sola-Carvajal A., García-Carmona F., Sánchez-Ferrer A. (2011). Molecular characterization of a novel N-acetylneuraminate lyase from *Lactobacillus plantarum* WCFS1. Appl. Environ. Microbiol..

[65] Boutin S., Alburaki M., Mercier P.L., Giovenazzo P., Derome N. (2015). Differential gene expression between hygienic and non-hygienic honeybee (*Apis mellifera* L.) hives. BMC Genom..

[66] Salmena L., Poliseno L., Tay Y., Kats L., Pandolfi P.P. (2011). A ceRNA hypothesis: The Rosetta Stone of a hidden RNA language?. Cell.

[67] Huang Y., Pang Y., Xu Y., Liu L., Zhou H. (2024). The identification of regulatory ceRNA network involved in *Drosophila* Toll immune responses. Dev. Comp. Immunol..

[68] Robinson G.E. (1992). Regulation of division of labor in insect societies. Annu. Rev. Entomol..

[69] Kim D., Langmead B., Salzberg S.L. (2015). HISAT: A fast spliced aligner with low memory requirements. Nat. Methods.

[70] Langmead B., Trapnell C., Pop M., Salzberg S.L. (2009). Ultrafast and memory-efficient alignment of short DNA sequences to the human genome. Genome Biol..

[71] Pertea M., Pertea G.M., Antonescu C.M., Chang T.C., Mendell J.T., Salzberg S.L. (2015). StringTie enables improved reconstruction of a transcriptome from RNA-seq reads. Nat. Biotechnol..

[72] Kang Y.J., Yang D.C., Kong L., Hou M., Meng Y.Q., Wei L., Gao G. (2017). CPC2: A fast and accurate coding potential calculator based on sequence intrinsic features. Nucleic Acids Res..

[73] Mistry J., Bateman A., Finn R.D. (2007). Predicting active site residue annotations in the Pfam database. BMC Bioinform..

[74] Sun L., Luo H., Bu D., Zhao G., Yu K., Zhang C., Liu Y., Chen R., Zhao Y. (2013). Utilizing sequence intrinsic composition to classify protein-coding and long non-coding transcripts. Nucleic Acids Res..

[75] Wen M., Shen Y., Shi S., Tang T. (2012). miREvo: An integrative microRNA evolutionary analysis platform for next-generation sequencing experiments. BMC Bioinform..

[76] Friedländer M.R., Mackowiak S.D., Li N., Chen W., Rajewsky N. (2012). miRDeep2 accurately identifies known and hundreds of novel microRNA genes in seven animal clades. Nucleic Acids Res..

[77] Robinson M.D., McCarthy D.J., Smyth G.K. (2010). edgeR: A Bioconductor package for differential expression analysis of digital gene expression data. Bioinformatics.

[78] Love M.I., Huber W., Anders S. (2014). Moderated estimation of fold change and dispersion for RNA-seq data with DESeq2. Genome Biol..

[79] Kopp F., Mendell J.T. (2018). Functional Classification and Experimental Dissection of Long Noncoding RNAs. Cell.

[80] Bao Z., Yang Z., Huang Z., Zhou Y., Cui Q., Dong D. (2019). LncRNADisease 2.0: An updated database of long non-coding RNA-associated diseases. Nucleic Acids Res..

[81] Enright A.J., John B., Gaul U., Tuschl T., Sander C., Marks D.S. (2003). MicroRNA targets in Drosophila. Genome Biol..

[82] Yu G., Wang L.G., Han Y., He Q.Y. (2012). clusterProfiler: An R package for comparing biological themes among gene clusters. OMICS.

[83] Shannon P., Markiel A., Ozier O., Baliga N.S., Wang J.T., Ramage D., Amin N., Schwikowski B., Ideker T. (2003). Cytoscape: A software environment for integrated models of biomolecular interaction networks. Genome Res..

[84] Livak K.J., Schmittgen T.D. (2001). Analysis of relative gene expression data using real-time quantitative PCR and the 2^−ΔΔCT^ Method. Methods.

